# Identification and characterization of the antiplasmodial activity of Hsp90 inhibitors

**DOI:** 10.1186/s12936-017-1940-7

**Published:** 2017-07-19

**Authors:** Claribel Murillo-Solano, Chunmin Dong, Cecilia G. Sanchez, Juan C. Pizarro

**Affiliations:** 1 Department of Tropical Medicine, School of Public Health and Tropical Medicine, Tulane University, New Orleans, LA USA; 2 Section of Pulmonary Diseases, Critical Care and Environmental Medicine, Department of Medicine, Tulane University Health Sciences Center, New Orleans, LA USA; 3 Vector-Borne Infectious Diseases Research Center, Tulane University, New Orleans, LA USA

**Keywords:** HSP90, Inhibitors, Repurposing, Drug development, Anti-malarial compounds

## Abstract

**Background:**

The recent reduction in mortality due to malaria is being threatened by the appearance of *Plasmodium falciparum* parasites that are resistant to artemisinin in Southeast Asia. To limit the impact of resistant parasites and their spread across the world, there is a need to validate anti-malarial drug targets and identify new leads that will serve as foundations for future drug development programmes targeting malaria. Towards that end, the antiplasmodial potential of several Hsp90 inhibitors was characterized. Because, the Hsp90 chaperone has been suggested as a good drug target against multiple parasitic infections including malaria.

**Results:**

Chemically diverse sets of Hsp90 inhibitors, evaluated in clinical trials as anti-cancer agents, were tested against the malaria parasite. Most of the compounds showed strong antiplasmodial activity in growth inhibition assays against chloroquine sensitive and resistant strains. There was a good agreement between the compound in vitro anti-parasitic activity and their affinity against the *Plasmodium* chaperone. The two most potent Hsp90 inhibitors also showed cytocidal activity against two *P. falciparum* strains. Their antiplasmodial activity affected all parasite forms during the malaria blood cycle. However, the compounds activity against the parasite showed no synergy when combined with anti-malarial drugs, like chloroquine or DHA.

**Discussion:**

The Hsp90 inhibitors anti-parasitic activity correlates with their affinity to their predicted target the *P. falciparum* chaperone Hsp90. However, the most effective compounds also showed high affinity for a close homologue, Grp94. This association points to a mode of action for Hsp90 inhibitors that correlate compound efficacy with multi-target engagement. Besides their ability to limit parasite replication, two compounds also significantly impacted *P. falciparum* viability in vitro. Finally, a structural analysis suggests that the best hit represents a promising scaffold to develop parasite specific leads according.

**Conclusion:**

The results shown that Hsp90 inhibitors are lethal against the malaria parasite. The correlation between biochemical and in vitro data strongly supports Hsp90 as a drug target against the malaria parasite. Furthermore, at least one Hsp90 inhibitor developed as anticancer therapeutics could serve as starting point to generate *P. falciparum*-specific lead compounds.

**Electronic supplementary material:**

The online version of this article (doi:10.1186/s12936-017-1940-7) contains supplementary material, which is available to authorized users.

## Background

Malaria is a deadly disease that kills over half a million people each year [1, 2]. Current control efforts rely on the use of insecticide-treated bed nets to prevent infection, and chemotherapy to treat *Plasmodium* infected individuals [3]. Over the last decade these measures have been efficacious in significantly reducing malaria’s incidence and mortality [4]. However, these gains are being threatened by the appearance of artemisinin resistant parasites [1, 3]. Artemisinin-based combination therapy (ACT)—in which the fast acting artemisinin or one of its derivatives is combined with an additional slow-clearance anti-malarial drug—is the first and last line of defense against the parasite. This dire situation highlights the need to investigate and identify new potential drug targets and drug leads (chemical scaffolds) to develop novel anti-malarial drugs [5].

The heat shock protein 90 (Hsp90) is an essential molecular chaperone in eukaryotes that has been suggested as a potential drug target against protozoan parasites [6]. The chaperone is a homodimer associated via its C-terminal or dimerization domains, and its middle or substrate-binding domain assists in the final folding stage of nascent proteins [7]. These Hsp90 substrates are called clients and represent ~5% of the cell proteome, including protein kinases, phosphatases and transcription factors that require chaperone assistance to reach their active state [8]. Many of the Hsp90 clients are essential proteins involved in multiple cell regulatory processes and required for the survival of the cell [9]. This keystone position of Hsp90 explains the essentiality of this chaperone in eukaryotes. ATP provides the energy necessary for the folding of the client protein, and a series of structural rearrangements associates the binding of the client with the hydrolysis of the phosphonucleotide. The Hsp90 N-terminal domain is a nucleotide-binding domain [10], but the ATPase activity requires a catalytically active residue located in the middle domain [7]. The conformational changes linking the ATPase and chaperone activities are called the Hsp90 catalytic cycle [7]. This connection allows ATP competitive inhibitors to limit its chaperone function, thereby affecting the concentration of Hsp90 clients [11]. These Hsp90 inhibitors are toxic to cells and organisms that heavily depend on the Hsp90 chaperone function, such as cancer cells or parasites [12, 13].

Hsp90 inhibitors possess several characteristics that make them promising drug leads against *Plasmodium falciparum*. First, these inhibitors have already been shown to limit parasite growth both in vitro and in the mouse malaria model [14–17]. Second, Hsp90 is an active target as anticancer therapy with several inhibitors already in clinical development [18, 19], thus it opens the possibility of repurposing these compounds as anti-malarial drug leads [15]. Finally, Hsp90 inhibition in drug resistant fungi reverts the phenotype from being resistant to sensitive, associating the chaperone activity with resistant traits [20, 21]. This is a significant observation since recent reports have linked artemisinin resistance with an enhanced stress response [22].

**Fig. 1:**
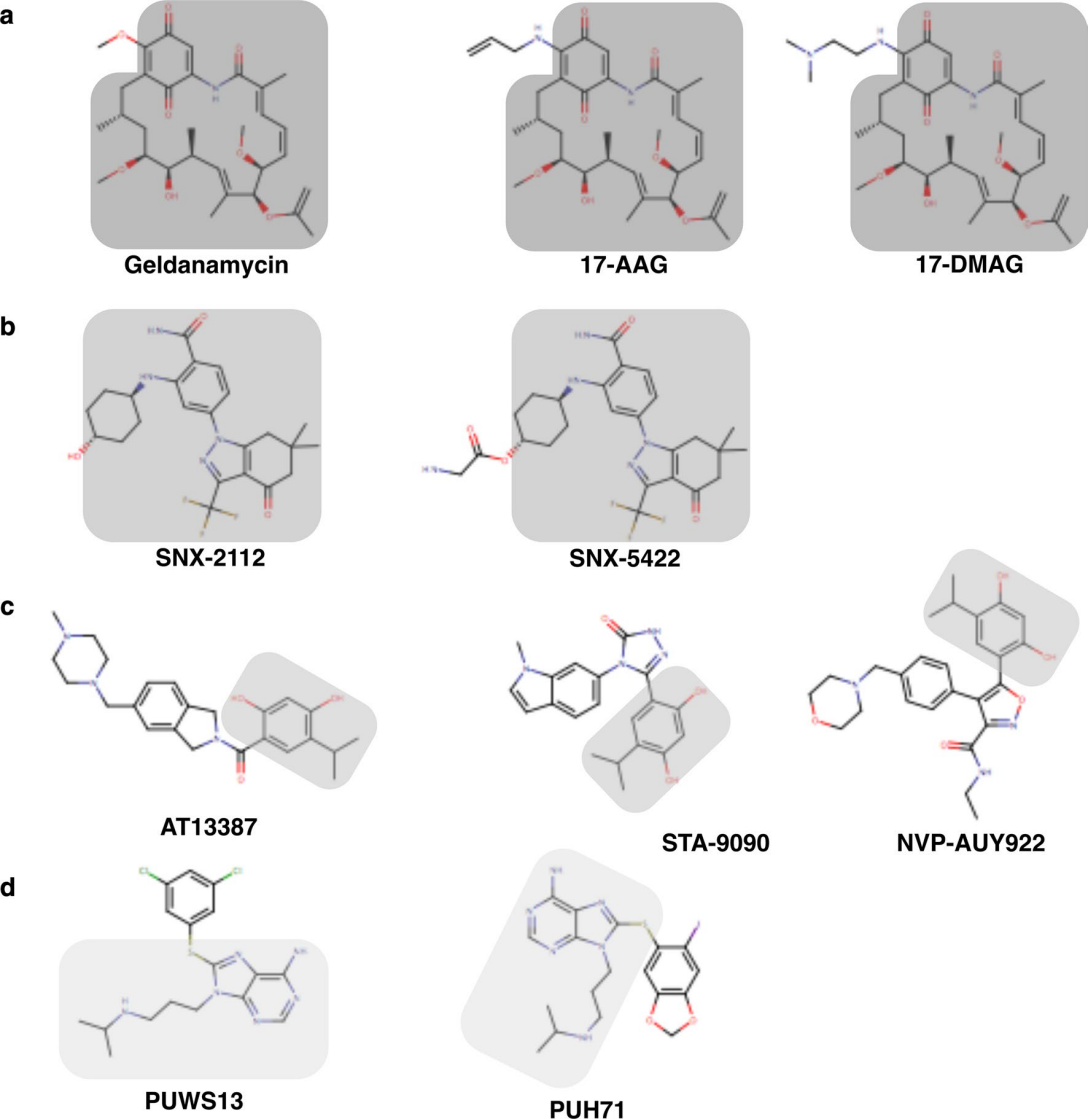
Chemical structures of Hsp90 inhibitors tested against *Plasmodium falciparum*. The compounds are arranged by scaffold, ansamycins (**a**), benzamides (**b**), resorcinol (**c**) and purines (**d**). The common part of each scaffold is *shaded*

These arguments make Hsp90 a very attractive target to identify potential drug leads against *P. falciparum*. Towards that end, a set of Hsp90 inhibitors were evaluated for their anti-parasitic activity. Compound set was selected to include all possible scaffolds that have been in clinical development as anticancer agents (Fig. 1). This piggybacking limits the risk of selecting compounds with poor pharmacological properties (e.g., poor solubility and toxicity) [23]. It also takes advantage of the information associated with the clinical development as anticancer agents, such as dosage and pharmacokinetic parameters. The Hsp90 inhibitors IC_50s_ were determined against two strains of *P. falciparum*, one chloroquine sensitive (3D7) and one resistant (W2), using a growth inhibition assay. Also, the inhibitors affinity (*K*_*d*_) for the *Plasmodium* chaperone was measured. For the most promising compounds, their cytotoxic effects against, both host cells and the malaria parasites were established. Additionally, the activity of these promising Hsp90 inhibitors was evaluated in combination with known anti-malarial drugs to identify potential synergistic effects. The results strongly support Hsp90 inhibition as a target to develop new anti-malarial compounds. In summary, a promising scaffold was identified that shows great antiplasmodial activity and has the potential to develop *P. falciparum* specific inhibitors.

## Methods

### Parasite cultures

The *P. falciparum* chloroquine sensitive 3D7 and chloroquine resistant W2 strains were maintained in continuous culture in RPMI-1640 medium (Gibco, Grand Island, NY) supplemented with 10% heat-inactivated non-immune AB human serum, 26 mg/L of hypoxanthine (Sigma-Aldric, St. Louis, MO) and 10 mg/L of Gentamicin (Gibco). A 50% suspension of non-infected O positive erythrocytes was added every 2 days to adjust haematocrit to 5%. Parasite cultures were maintained at 37 °C in 5% O_2_ and 5% CO_2_ atmosphere with daily changes of culture media.

### Hsp90 inhibitors

The Hsp90 inhibitors selected belong to three different scaffolds (Fig. 1): first, the ansamycin represented by geldanamycin [24], 17-AAG [25] and 17-DMAG [26]; second, the benzamides included SNX-5422 and SNX-2112 [27]; third, the resorcinol that included AT13387 [28], NVP-AUY922 [29], and STA-9090 [30]; and fourth, the purine scaffold that included compounds PUH71 and PUSW13 [31, 32]. All compounds were used from stock solutions ranging from 10 to 30 mM in 100% DMSO. The Hsp90 inhibitors and dihydroartemisinin (DHA) used in the study were commercially available and purchased from a single vendor (Selleck Chemicals, Houston, TX). Chloroquine (CQ) (Sigma-Aldrich, St. Louis, MO) was used from a stock solution in water.

### Growth inhibition assay

A synchronized *P. falciparum* culture, with over 80% ring forms, was used in the growth inhibition assay. Each strain was assayed at 0.5% parasitaemia and 1.5% haematocrit. A 190 μL aliquot of parasite culture was dispensed per well to microplates that were pre-dispensed with the compounds at various dilutions. A 10 μL aliquot of 11 dilutions per compound had been previously dispensed into a 96-well flat-bottom, in duplicate. Dilutions of Hsp90 inhibitors and DHA were prepared in water with a final concentration of DMSO below 0.05% (v/v). Preliminary assays determined that this DMSO concentration did not impact *P. falciparum* growth when compared to non-exposed cultures.

Control parasites and parasites exposed to CQ dilutions did not contain any DMSO. The drug/inhibitor dilutions cover the following ranges: CQ from 3.12 nM to 3.2 µM; DHA from 0.125 to 128 nM; Geldanamycin, 17-AAG, 17-DMAG, SNX-5422, AT13387, NVP-AUY922 and STA-9090 from 4.88 nM to 5 µM; SNX-2112 from 9.8 nM to 10 µM; and PUH71 and PUWS13 from 100 nM to 10 µM. The microplates were incubated at 37 °C in a controlled atmosphere (5% O_2,_ 5% CO_2_ Nitrogen balanced) for 72 h. After incubation, the plates were subject to a freeze–thaw cycle (−80 °C) and the parasite growth was quantified by the SYBR Green I method as previously described [33]. Briefly, 100 µL of homogenized parasite culture was transferred into 96-well black plates. 100 µL of SYBR Green lysis buffer (2× SYBR Green I, 100 mM Tris–HCl pH 7.5, 10 mM EDTA, 0.016% Saponin and 1.6% Triton X-100) were added to each well. The plates were incubated at room temperature in darkness for 1 h. The fluorescence signal was read in a Synergy HT (Biotek Instruments, Inc., Winooski, VT) plate reader with 485_Ex_/520_Em_ nm.

The 50% inhibitory concentrations (IC_50_) for each compound were calculated using the online program ICEstimator 1.2 [34]. IC_50_ values were used in the analysis only if the parasite growth index, determined by the ratio of fluorescence units (FU) between the parasite grown without compound versus signal at maximum compound concentration, was equal to or higher than two. Growth inhibition experiments were conducted at least three times per compound (biological replicates, n ≥ 3). The IC_50_ differences for each inhibitor between the two *P. falciparum* strains were compared with a *t* test. The statistical analysis was performed with the software Prism v5.01 (GraphPad Software, San Diego, CA).

### Drug cytotoxicity assay

Primary normal human lung fibroblasts (NHLFs) were seeded on 24-well plates and cultured in FGM-2 media (LONZA, Basel, Switzerland) until they reached 80% confluency. The cells were treated with NVP-AUY922 and 17-DMAG at varying concentration from 0.5 to 10 µM in 0.5 mL FGM-2 media for 24 h. The colorimetric tetrazole based MTT assay was performed using an in vitro Toxicology Assay Kit (Sigma) according to manufacturer’s instructions. Briefly, 50 µL of MTT dye was added into the media and the cells were returned to the incubator for 3 h. Then 500 µL of solubilization solution was added to dissolve the MTT formazan crystals. The absorbance at a wavelength of 570 nm was measured by spectrophotometer in triplicate. The background absorbance at 690 nm was also measured and subtracted from the Abs_570_ measurement. The drug cytotoxicity assay was performed in triplicate.

### Recrudescence assay

The assay is a modification of the previously reported bulk recrudescence assay [35]. For each strain, a non-synchronized culture with a ~2% parasitaemia and 5% haematocrit was exposed to three concentrations of the Hsp90 inhibitor alone or in combination with 100 mM DHA for 48 h. The Hsp90 inhibitor concentrations were 1.5, 3 and 6 µM for 17-AAG; 0.3, 0.6 and 1.2 µM for 17-DMAG; and 0.18, 0.36 and 0.72 µM for NVP-AUY922. These concentrations represented roughly 5, 10 and 20 times the average IC_50_ for 17-AAG, and 2, 4 and 8 times for 17-DMAG, and 2, 5 and 9 times for NVP-AUY922. After drug exposure, the medium containing the drug was removed and the cultures were washed twice with compound-free media, and resuspended in RPMI to adjust haematocrit to 5%. The medium was changed every day and red blood cells were added every 2 days to adjust the haematocrit. The cultures were monitored daily until they reached 10% parasitaemia, which represented the end point of the experiment. Culture parasitaemia was determined by scoring >2000 erythrocytes in Giemsa-stained thin blood films as parasitized or not. The median number of days to reach this level of parasitaemia was reported for all the experiments. All experiments were conducted in triplicate; each replica was conducted on different days. The differences in the recovery times between control cultures to those exposed to Hsp90 inhibitors were analysed using the non-parametric statistic Kruskal–Wallis test. If the overall test was significant then Dunn’s multiple comparison tests were used to identify significant differences among pair comparisons. The same statistical analysis was applied when comparing *P. falciparum* cultures exposed to DHA alone versus DHA in combination with Hsp90 inhibitors. A two-way ANOVA was used to compare effects of *P. falciparum* strain and compound concentrations for the each of the two geldanamycin derivatives. The statistical analyses were performed with the software Prism v6 (GraphPad Software, San Diego, CA).

### Parasite stage specific effect

Synchronized *P. falciparum* cultures were used to determine the susceptibility of different parasite stages to Hsp90 inhibitors. Three synchronized cultures per strain, with a majority of the parasites in the ring (0–26 h post red blood cell infection), trophozoite (26–38 h) or schizont (38–48 h) stages respectively (Fig. 3). The cultures were assayed at 0.5% parasitaemia and 1.5% hematocrit under conditions previously described. The cultures were exposed to one concentration of the Hsp90 inhibitor for 24 h. The Hsp90 inhibitor concentrations were 6 µM for 17-AAG; 1.2 µM for 17-DMAG; and 0.72 µM for NVP-AUY922. For each three exposed the cultures and the control for both strains 3D7 and W2, the parasitaemia and the developmental stage of the parasites were recorded. Before and after drug exposure, Giemsa-stained blood smears were scored by microscopy examination counting 100 infected erythrocytes per culture, and the proportion of each developmental stage was calculated.

### Drug combination assays

Four compound combinations were evaluated against both strains of *P. falciparum* in an 8 × 8 dose matrix combination between an Hsp90 inhibitor (NVP-AUY922 and 17-DMAG) and antimalarial compound (DHA and CQ). Each compound IC_50_ established the midpoint of the range of concentrations tested. Seven twofold dilutions were included for each compound in a 96-well plate (see Additional file 1: Figure S2 for plate design and concentrations used). Each matrix included a control without drug, and two rows for Hsp90 inhibitor and the antimalarial drug alone. The combination assays were setup similarly to the previously described growth inhibition assays, with the following modification: plates included a 20 µL predispensed compound mixture and a 180 µL parasite suspension. The parasite growth was quantified using the SYBR green method as previously described. The interaction between the Hsp90 inhibitor and antimalarial compound was analysed using a Loewe additivity model available in the Chalice Analyzer Online software (Horizon Discovery Group, Cambridge, UK).

### Recombinant protein production and purification

Full-length human recombinant HSP90α was purchased (Cayman Chemicals, MI), while the malaria recombinant parasite proteins were generated according to the following protocol. *P. falciparum* HSP90 (PF3D7_0708400) residues 1-212 and GRP94 (PF3D7_1222300) residues 72-284 were amplified from cDNA by PCR, ligated into a pET15 expression vector containing a C-terminal hexahistidine tag and subsequently transformed into *E. coli* Rosetta2 (Novagen) competent cells. Successful transformants were grown in LB overnight at 37 °C, these cultures inoculate a 1 L TB culture. Growth at 37 °C continued until the OD_600_ reached ~1.0, whereupon the temperature was lowered to 18 °C and protein expression was induced by the addition of 1 mM isopropyl β-d-thiogalactopyranoside (IPTG). Overnight post-induction cells were collected by centrifugation and resuspended in binding buffer [50 mM Hepes pH 7.5, 500 mM NaCl, 1 mM benzamidine, and 1 mM phenylmethanesulfonyl fluoride (PMSF)]. The cells were lysed by adding chemical lysis buffer (2% CHAPS, 0.1 mg/mL lysozyme and 0.5 U/mL benzonase), the lysate was clarified by centrifugation. The supernatant was loaded on to a nickel-nitrilotriacetic acid column pre-equilibrated with binding buffer (50 mM Hepes pH 7.5, 500 mM NaCl). Following a wash step with binding buffer containing 5 mM imidazole, the bound protein was eluted with elution buffer (50 mM Hepes pH 7.5, 500 mM NaCl, 250 mM imidazole). The eluate was then loaded on to a 16/60 Superdex 200 size exclusion column pre-equilibrated with 50 mM Hepes pH 7.5 and 500 mM NaCl. Target protein-containing fractions were pooled and concentrated using centrifugal filter units (Amicon). The final protein products and samples for different steps in the purification procedures were evaluated in SDS-Page gels.

### Biacore analysis

SPR measurements were performed on Biacore T200 instrument (GE Healthcare) at 25 °C. Purified *P. falciparum* Hsp90 and PfGRP94 N-terminal domain, and full-length human Hsp90α were immobilized on a CM5 sensorchip using NHS/EDC coupling following the manufacturer protocol to a level of <10,000 RUs, a reference surfaces without immobilized proteins served as a control for nonspecific binding and refractive index changes. Seven different concentrations of the ligands between 0.4 nM and 1 mM, were injected in triplicate over the sensor chip at 30 μL/min in random order. The running buffer was 10 mM HEPES, pH 7.4, 150 mM NaCl, 0.005% P20, 1% DMSO and 2 mM MgCl_2_. Buffer alone injections were used as blanks. The sensor surface was regenerated between injections with a short injection of a buffer 10 mM HEPES, pH 7.4, 150 mM NaCl, 0.005% P20 and 3 mM EDTA. The Biacore responses recorded during the 30 s of the injection were used to estimate the association constant (*k*_*on*_) and the 300 s after the injection were used to estimate the dissociation constant (*k*_*off*_). All kinetic constants were estimated using double subtracted sensorgrams using BiaEvaluation Software (GE Healthcare), the kinetic constants were used to calculate the dissociation constant (*K*_*d*_).

### Molecular docking

The *P. falciparum* crystallographic structure in complex with ADP (PDB entry 3K60 [36]) was used in the docking procedure to examine the potential binding mode of NVP-AUY922. The protein coordinates of the parasitic structure were used in combination with the ligand coordinates extracted from the human Hsp90–NVP-AUY922 crystallographic structure (PDB entry 2VCI [29]). The docking space was defined manually using MGL tools [37] and the docking procedure implemented in VINA was employed [38]. The docking solutions were visually inspected and the top solution was selected and compared with the human crystallographic structure.

## Results

### Malaria parasite susceptibility to Hsp90 inhibitors

The eight Hsp90 inhibitors that were tested strongly suppressed parasite growth and six of them showed an IC_50_ in the submicromolar range (Table 1; Additional file 1: Figure S1 for normalized dose response graphs). As expected, a strain specific susceptibility to the anti-malarial drug CQ was observed, but also significant differences were observed between strains for DHA and the Hsp90 inhibitors: 17-DMAG, 17-AAG, SNX-2112 and PUWS13 (*t* test, P < 0.05). The remaining five chaperone inhibitors tested showed no significant differences in their growth inhibition capabilities between the two strains (3D7 and W2).

**Table 1:** Growth inhibitory activity of Hsp90 inhibitors against *Plasmodium falciparum*

		3D7			W2		
		IC_50_ (nM)	CI 95%	IC_90_ (nM)	IC_50_ (nM)	CI 95%	IC_90_ (nM)
CQ	*	37.9	35.7–40.0	67.2	128.7	118.4–138.9	389.7
DHA	*	1.2	1.1–1.3	5.2	0.5	0.4–0.6	1.8
Ansamycin							
Geldanamycin		214.8	180.1–249.5	1700.0	200.2	153.3–247.1	2625.3
17-DMAG	*	118.1	109.4–126.7	286.6	169.5	153.3–185.7	541.9
17-AAG	*	387.5	277.7–497.4	8843.3	161.3	100.1–222.4	9569.7
Resorcinol							
AT13387		665.0	577.2–752.9	4570.9	530.7	418.2–643.3	5369.2
NVP-AUY922		75.5	67.2–83.7	646.1	75.4	64.7–86.0	460.2
STA-9090		1254	982.7–1525	4110.6	1294	488.1–2101	>10.000
Benzamides							
SNX-5422		2136	161.9–2661	4322.6	2008	1155–2860	6648.4
SNX-2112	*	665.0	577.2–752.9	4570.9	1702	697.4–2707	>10.000
Purines							
PUH71		3920	3410–4430	>10.000	4420	3930–4910	>10.000
PUWS13	*	7000	6180–7820	>10.000	3880	3400–4360	9060

IC_50_ and IC_90_ values and their confidence interval (CI) at 95% confidence level are presented

* Significant differences between strains (two-tailed, t test *P* < 0.05)

Among the tested compounds, the benzamide and purine scaffolds were the least effective at inhibiting parasite growth in vitro, and their IC_50s_ were relatively high compared to the other compounds tested (Table 1). The natural product geldanamycin and its derivatives inhibited the parasite growth in the submicromolar range. 17-DMAG was the top hit of the ansamycins tested with an IC_50_ of 118 and 169 nM against 3D7 and W2 respectively. This confirmed the strong antiplasmodial activity of the geldanamycin scaffold [16]. However, the top Hsp90 inhibitor against *P. falciparum* was NVP-AUY922, with an IC_50_ ~75 nM, similar to that of chloroquine against 3D7. Encouragingly, this compound was equally effective against the CQ sensitive and CQ resistant *P. falciparum* strains.

### Parasite survival after Hsp90 inhibitor exposure

**Fig. 2:**
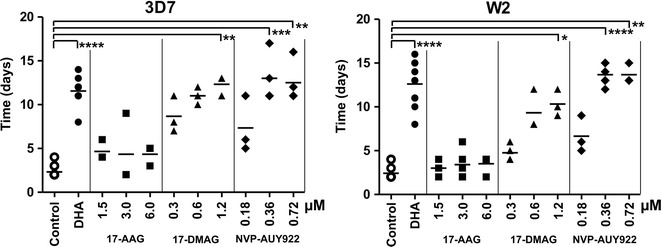
The recovery time of *P. falciparum* cultures exposed to Hsp90 inhibitors. A dot plot of the number of days required to reach 10% parasitaemia after 48 h exposure to the compounds (n ≥ 3 independent replicates). A *horizontal bar* for each sample indicates the median. Two controls are included for each strain, 3D7 (*left*) and W2 (*right*), a no drug control and a drug control (100 nM DHA). Significant differences *P < 0.05, **P < 0.005, ***P < 0.001 and ****P < 0.0001, Kruskal–Wallis Dunn’s post hoc test

The recrudescence assay measures the recovery time of parasite cultures exposed to different concentrations of Hsp90 for 48 h. Fast recovery times imply that the compound killed fewer parasites and longer times are associated with a large proportion of the parasites in culture being eliminated by the drug. Three compounds with submicromolar IC_50s_ from the growth inhibition assay, NVP-AUY922, 17-DMAG and 17-AAG, were evaluated in the recrudescence assay. As a benchmark of cytocidal activity against the malaria parasite a time-limited exposure to a high concentration of DHA was used [39], because of its known cytotoxic effect and fast acting antiplasmodial activity [40]. The control cultures exposed to 100 nM DHA for 48 h recovered to 10% parasitaemia in 12 and 13 days (median values), for 3D7 and W2 respectively; while non-exposed cultures reached the same parasite density in 2.3 days for 3D7 and 2.4 days for W2 (Fig. 2). The three Hsp90 inhibitors showed great variability in the recrudescence assay. The most active compound against both 3D7 and W2 was NVP-AUY922. The two highest concentrations assayed 5 and 9 times its IC_50_ (0.36 and 0.72 µM) significantly delayed the parasite recovery when compared to the untreated parasites (Dunn’s multiple comparison test, P < 0.005). 17-DMAG was only significantly different from the control parasite cultures when assayed at a concentration ~8 times its average IC_50_ against *P. falciparum*. The least effective compound in the recrudescence assays was 17-AAG. None of the concentrations tested, ranging from 4 to 16 and 9 to 36 times its IC_50_ against *P. falciparum* 3D7 and W2, respectively, significantly delayed the culture recovery time when compared to the control.

The geldanamycin derivatives 17-AAG and 17-DMAG differed significantly in their IC_50s_ against *P. falciparum* 3D7 and W2 strains (*t* test, P < 0.05). However, only 17-DMAG showed significant differences in the recrudescence assay; 3D7 was more sensitive than W2 to this derivative (two-way ANOVA, F(1,13) = 12.42, P < 0.005 between strains; F(2,13) = 15.59, P < 0.001 between inhibitor concentrations and F(2,13) = 0.99 interaction P > 0.05). This result highlights the enhanced sensitivity to this compound by *P*. *falciparum* 3D7.

### Parasite stage susceptibility to Hsp90 inhibitors

**Fig. 3:**
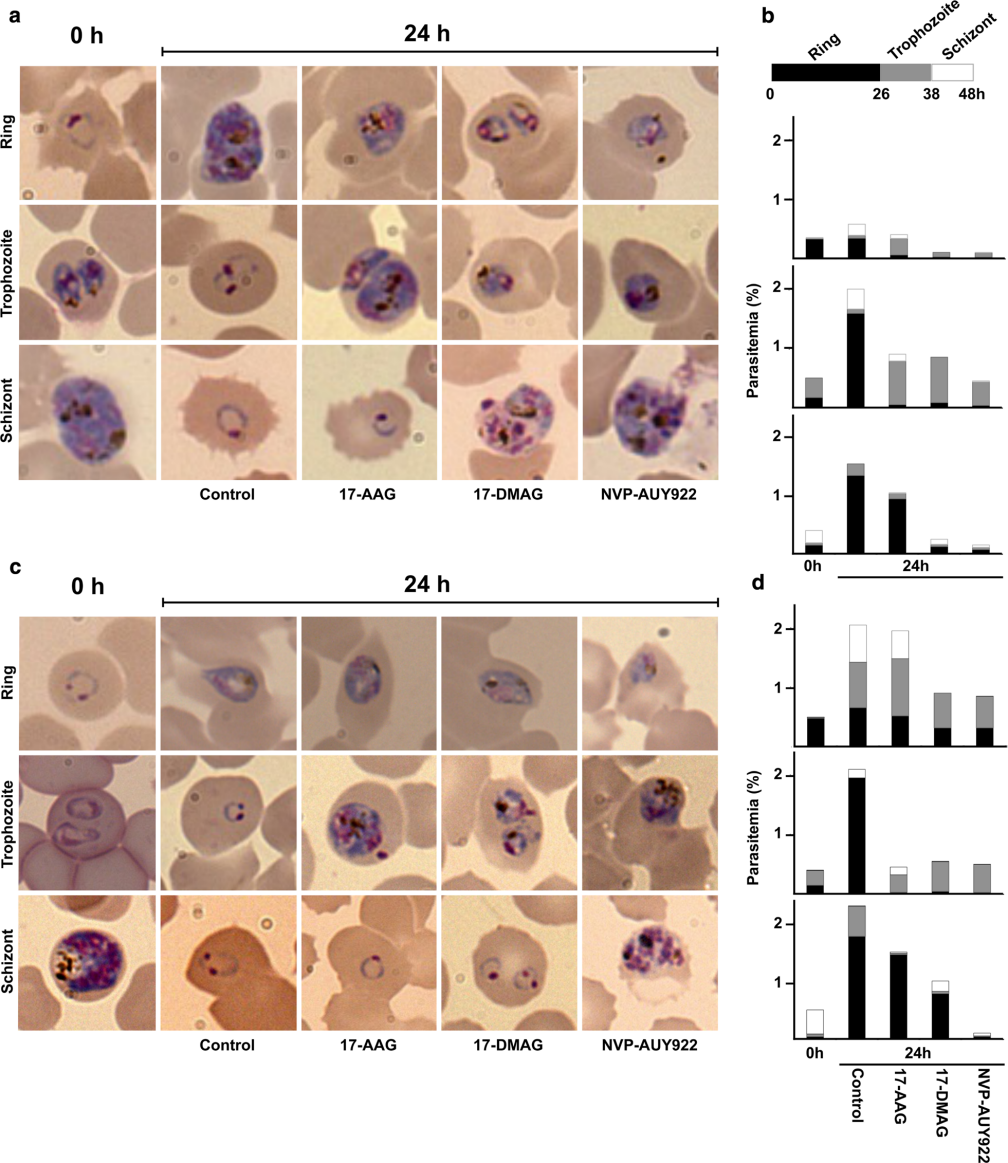
Morphology of *P. falciparum* 3D7 (**a**, **b**) and W2 (**c**, **d**) parasites exposed to different Hsp90 inhibitors. Ring, Trophozoite or Schizont synchronized cultures were exposed to three compounds 17-AAG (6 µM), 17-DMAG (1.2 µM) and NVP-AUY922 (0.72 µM) for 24 h. The time 0 h represents the starting point of the cultures, and each *photo* represent the most abundant parasite stage observed post exposure or control. For each culture 100 infected erythrocytes were counted; a *bar graph* indicates the parasitaemia, and *different shades* indicates the proportion of different parasite developmental stages (**b**, **d**). Photos of Giemsa stained thin films at ×100 magnification

Compound efficacy against the malaria parasite is correlated to its ability to affect different developmental stages of *P. falciparum* [41]. Compound efficacy was evaluated against three different stages of the parasite: ring, trophozoite and schizont. Synchronous parasite cultures at different developmental stages were exposed to the highest concentration of 17-AAG, 17-DMAG and NVP-AUY922 previously evaluated in the recrudescence assay. The parasitaemia and the morphology of the malaria parasites after exposure to the three Hsp90 inhibitors for 24 h showed some differences associated with the susceptibility of the developmental stage and the compound (Fig. 3). The common feature was that all compounds were effective against trophozoites from both strains 3D7 and W2. 17-AAG showed no effect against the ring stage in both CQ sensitive or resistant *P. falciparum* strains, and limited effect against schizonts. These observations are consistent with the results from the recrudescence assay that showed no growth delay for this Hsp90 inhibitor. Conversely, 17-DMAG and NVP-AUY922 were effective against all parasite stages. As seen in the recrudescence assay, both compounds were more effective against 3D7 than to W2 (Fig. 2). Finally, NVP-AUY922 was the most effective compound, affecting all stages in both strains.

### The antiplasmodial effect of anti-malarial drug–Hsp90 inhibitor combinations

**Fig. 4:**
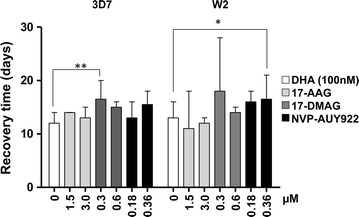
The recovery time of *P. falciparum* cultures exposed to Hsp90 inhibitors in combination with DHA. Each *bar* represents the median number of days required for the parasite cultures to reach 10% parasitaemia (n ≥ 3 independent experiments). Statistically significant differences (*P < 0.05 and **P < 0.005) were determined by Kruskal–Wallis Dunn’s post hoc test

The recrudescence assay was also used to evaluate antimalarial–Hsp90 inhibitor combinations against *P. falciparum*. As for the single agent assays, the same three Hsp90 inhibitors were tested—17-AAG, 17-DMAG and NVP-AUY922–at two different concentrations in combination with a DHA at a single concentration (100 nM). The culture recovery times were compared against parasite cultures exposed to DHA alone. The Hsp90 inhibitors concentrations were ~5 and 10 times the IC_50_ for 17-AAG, and 2–5 times the IC_50_ for 17-DMAG and NVP-AUY922. The results were concordant with the single agent results (Fig. 4). 17-AAG in combination with DHA did not affect the recovery time of either *P. falciparum* strain. The second geldanamycin derivative, 17-DMAG was more effective in combination against *P. falciparum* 3D7 but only at one of the lowest concentrations (Dunn’s multiple comparison test, P < 0.005). The 17-DMAG–DHA combination was not significantly more toxic to W2 when compared to the effect of the antimalarial drug alone. The combination results reinforced the observation that this geldanamycin derivative is more potent against 3D7. Finally, both strains showed an increase in the time required to reach 10% parasitaemia when treated with the highest dose of the NVP-AUY922. The increase was almost five times its IC_50_ in combination with DHA. However, the difference was only significant in the case of *P. falciparum* W2 (Dunn’s multiple comparison test, P < 0.05).

Additionally, interactions between anti-malarial drugs and Hsp90 inhibitors were evaluated, using a matrix combination approach. The two most potent Hsp90 inhibitors against *P. falciparum*, 17-DMAG and NVP-AUY922, were combined with two anti-malarial drugs DHA and CQ. The *P. falciparum* growth inhibition was evaluated for these four drug combinations using a Loewe additivity model to predict the effect of the drug-Hsp90 inhibitor combination. Overall, the combinations tested did not deviate from an additive effect. The additive effect was also evident in the isoboles (see Additional file 1: Figures S3, S4).

### Toxicity of Hsp90 inhibitors in human primary cells

The cytotoxic effect of 17-DMAG and NVP-AUY922 against primary human lung fibroblasts was determined. The Hsp90 inhibitors were assayed at concentrations up to 10 µM. A visual inspection did not reveal any morphological differences and cell densities were not noticeably different between the controls and the treated fibroblasts. In the case of NVP-AUY922, none of the concentrations tested significantly inhibited the growth of host cells compared to no drug controls. In the case of 17-DMAG, only the highest concentration (10 µM) was significantly different from the controls (two-tailed t test, P < 0.05). Since the growth inhibition for the human cells never exceeded 25%, this indicates an IC_50_ >10 µM (data not shown).

### HSP90 inhibitors affinity and selectivity towards the *Plasmodium falciparum* chaperone

Dissociation constants (*K*_*d*_) of recombinant ATP binding domains from *P. falciparum* Hsp90 and Grp94 towards the inhibitors tested in the growth inhibition assays were determined (Table 2). As a reference, the binding affinity of the chaperone-ADP interaction was measured, as expected the calculated *K*_*d*_ was in the micromolar range. Also, as it has been previously reported for Grp94 proteins, this parasite chaperone showed a higher affinity towards ADP than the cytosolic one [42]. The parasitic HSP90 bind inhibitors with high affinity, 7 out of the 10 compounds tested bind the *Plasmodium* protein with submicromolar *K*_*d*_ values (Table 2; see Additional file 1: Figures S5–S7 for SPR sensorgrams). The *P. falciparum* HSP90 chaperone showed high affinity for at least one compound from all the different scaffolds tested. The resorcinol-based compounds had only one compound with relatively low affinity (AT13387), while geldanamycin and its derivatives showed only one compound with high affinity (17-DMAG). As expected, there was little selectivity for the *Plasmodium* chaperone vis-à-vis its human counterpart; the notable exception was the purine scaffold, PUH71 and PUW13 affinities were over 1000-fold lower for the parasite chaperone. Finally, three compounds -17-DMAG, NVP-AUY922 and STA-9090- showed strong binding towards all the chaperones. Despite the fact that specificity was different between all three chaperones as determined by ranking of the tested compounds according to their *K*_*d*_s.

**Table 2:** Affinity of *P. falciparum* Hsp90 and Grp94, and human Hsp90α against ADP and known chaperone inhibitors

	*P. falciparum*								Human			
	Hsp90				Grp94				Hsp90α			
	K_d_ (µM)	k_a_ (1/Ms)		SE	K_d_ (µM)	k_a_ (1/Ms)		SE	K_d_ (µM)	k_a_ (1/Ms)		SE
		k_d_ (1/s)				k_d_ (1/s)				k_d_ (1/s)		
ADP	20.6	107	±	2.5 × 10^−1^	2.21	1360	±	4.3 × 10^1^	63.8	109	±	7.1 × 10^−1^
		0.002	±	6.5 × 10^−6^		0.003	±	4.9 × 10^−5^		0.006	±	2.5 × 10^−5^
Geldanamycin	3.64	529	±	1.2 × 10^2^	1530	346	±	8.6 × 10^0^	1110	393	±	1.1 × 10^2^
		0.002	±	4.4 × 10^−4^		0.529	±	5.6 × 10^−3^		0.435	±	4.5 × 10^−3^
17-DMAG	0.008	208,000	±	1.1 × 10^4^	0.023	53,000	±	5.4 × 10^4^	0.158	4600	±	1.4 × 10^4^
		0.001	±	7.7 × 10^−5^		0.001	±	5.5 × 10^−4^		0.007	±	7.3 × 10^−4^
17-AAG	4.52	371	±	6.1 × 10^1^	28.5	9470	±	1.4 × 10^3^	0.093	10,900	±	2.4 × 10^4^
		0.001	±	3.1 × 10^−4^		0.002	±	1.6 × 10^−4^		0.01	±	3.3 × 10^1^
AT13387	46.5	212	±	1.3 × 10^1^	2980	216	±	6.4 × 10^0^	61	570	±	3.3 × 10^1^
		0.009	±	8.5 × 10^−5^		0.643	±	5.1 × 10^−3^		0.348	±	2.6 × 10^−3^
NVP-AUY922	0.013	107,000	±	7.6 × 10^4^	0.009	438,000	±	3.5 × 10^4^	0.012	105,000	±	7.5 × 10^3^
		0.001	±	7.6 × 10^−4^		0.004	±	2.7 × 10^−4^		0.001	±	7.2 × 10^−5^
STA-9090	0.121	1.27	±	1.2 × 10^−1^	0.001	1.7x10^6^	±	3.0 × 10^5^	0.27	2080	±	1.2 × 10^2^
		1.5 × 10^−7^	±	2.8 × 10^−5^		0.003	±	3.1 × 10^−4^		0.0005	±	2.6 × 10^−5^
SNX-2112	0.183	20,300	±	6.5 × 10^−6^	627	4720	±	1.3 × 10^3^	0.001	2.0 × 10^6^	±	1.9 × 10^5^
		0.003	±	9.2 × 10^−5^		0.0006	±	1.0 × 10^−4^		0.002	±	2.5 × 10^−4^
PUH71	0.015	356,000	±	4.5 × 10^5^	6.43	5830	±	4.3 × 10^2^	0.262	270	±	4.3 × 10^0^
		0.005	±	4.9 × 10^−5^		0.006	±	7.7 × 10^−5^		0.007	±	6.5 × 10^−5^
PUWS13	0.069	598,000	±	1.5 × 10^5^	5600	149	±	2.4 × 10^0^	0.274	811	±	2.9 × 10^1^
		0.041	±	1.7 × 10^−3^		0.843	±	4.7 × 10^−3^		0.022	±	5.9 × 10^−5^

*K*_*d*_ values, and the corresponding kinetic constants (*k*_*a*_ and *k*_*d*_) and their standard errors (SE) are presented

## Discussion

The Hsp90 chaperone has been previously recognized as a potential antiplasmodial target [6]. But, previous studies have only explored a limited portion of Hsp90 inhibitors’ chemical diversity against the malaria parasite in vivo and in vitro [16, 43]. To identify an inhibitor scaffold with stronger antiplasmodial activity and favorable toxicity profile we have expanded the chemical diversity of the inhibitors tested as potential antimalarial leads. Four different chemical scaffolds were screened, each one represented by at least two compounds (Fig. 1). All of the screened compounds have been or are under clinical development, geldanamycin and its derivatives in the former category, and AT13387, NVP-AUY922, PUH71, SNX-2112 and STA9090 in the latter [44]. The information associated with their clinical development could be leveraged when exploring their antimalarial drug potential [45, 46].

All of the compounds tested inhibited the growth of the malaria parasite, further validating the potential of Hsp90 as a drug target against *Plasmodium* [16, 43, 47]. However, the possibility that the observed antiplasmodial activity is due to an off-target effect of the Hsp90 inhibitors could not be formally excluded. But such a situation is unlikely because of the proven selectivity for the tested compounds against Hsp90, and this is confirmed by the high affinity of the tested Hsp90 inhibitors to the *P. falciparum* chaperone (Table 2). Additionally, there is an association between *P. falciparum* Hsp90 *K*_*d*_ and IC_50_ values, Spearman correlation 0.38 and 0.45 for 3D7 and W2, respectively. But that correlation is lost when comparing *K*_*d*_ for the parasite GRP94 and IC_50_ values, Spearman correlation values of 0.14 and 0.01 for 3D7 and W2. Finally, it is further unlikely given the experimental validation of the geldanamycin scaffold as chaperone specific in both human and *P. falciparum* chaperones [24, 48], and for the lack of off-target effects against other ATP binding proteins like protein kinases [49]. Therefore, a more likely explanation for the activity of these inhibitors could be associated with the inhibition of additional members of the Hsp90 family, such as Grp94 or Trap-1 [50, 51]. The results support this hypothesis since the most active compounds against malaria parasite, 17-DMAG and NVP-AUY922, also showed the highest affinity for the two *Plasmodium* chaperones tested (Table 2).

The Hsp90 inhibitors with the lowest IC_50s_, nanomolar range, were two geldanamycin derivatives (17-AAG and 17-DMAG) and one resorcinol based compound (NVP-AUY922) (Fig. 1). The latter compound has the lowest IC_50_ reported for any chaperone inhibitor against the malaria parasite, and it is equally effective against both the CQ sensitive and CQ resistant *P. falciparum* strains. In contrast, the geldanamycin derivatives that showed significant differences between strains (Table 1): 17-AAG showed a lower IC_50_ against W2, while 17-DMAG was more potent towards 3D7. These differences in growth inhibition among the ansamycins can be attributed to compound variations, since the Hsp90 amino acid sequences are identical between 3D7 and W2. Such differences among geldanamycin derivatives have already been reported for other protozoan parasites [52].

The proposed mechanism of action of Hsp90 inhibitors is through cell cycle arrest [11, 53]; therefore low IC_50_ against the *Plasmodium* parasite could be caused by a strong growth inhibitory effect (cytostatic). Because cytostatic compounds are not suitable to develop anti-malarial drugs [54], the ability of some of these inhibitors to kill the malaria parasite (cytotoxic) was tested. The recrudescence assay identified 17-AAG as a cytostatic compound without cytotoxic activity (Fig. 2), since high concentrations of the compound showed no effect in limiting the growth of the parasite once the drug was removed. The lack of cytotoxicity of 17-AAG was unexpected because previous in vivo studies in the mouse malaria model have shown anti-parasitic effects for this compound [14, 16]. These findings could have resulted from a strong induction of the immune response against *Plasmodium* in the animals treated with the geldanamycin derivative [14]. On the contrary, both 17-DMAG and NVP-AUY922 showed strong cytotoxic activities against both strains of *P. falciparum* (Fig. 2). Additionally, the cytotoxic activities of the three Hsp90 inhibitors tested strongly correlate with their ability to limit the growth of the different parasite stages (Fig. 3). Hence, the robust antiplasmodial effect of NVP-AUY922 is associated with its impact throughout the parasite’s intraerythrocytic developmental cycle.

Monotherapy against malaria is widely discouraged due to the parasite’s ability to acquire drug resistant traits [55]. Using the recrudescence assay, the combination between the Hsp90 inhibitors and the anti-malarial drug, DHA, was evaluated. Some interactions were observed between DHA and the chaperone inhibitors, but the assay design did not allow us to evaluate of the nature of the drug-inhibitor interactions. To further characterize the interaction as synergistic, additive or antagonistic, the two most potent compounds were evaluated in combination with two anti-malarial drugs in a pair-wise dose–response combinatorial approach. Previous reports have identified synergistic [43, 56] or additive [57] interactions between the Hsp90 inhibitors and CQ or DHA, this study found that recorded growth inhibition rarely exceeded the expected value calculated from the effective dosage that was attained when combining both drugs (see Additional file 1: Figures S3, S4). This lack of concordance among studies can be explained by differences in methodologies used to evaluate drug–drug interactions. The results showed some slight synergistic interactions when the DHA–NVP-AUY922 combinations were analysed using an isobologram analysis. However, when the combination results were compared to a dosage-additive model (Loewe additivity), the two drugs showed no interaction and their combined effect did not deviate from the expected effect of adding the doses from the anti-malarial drugs and the Hsp90 inhibitor (equal effective dosage). The absence of synergy between the Hsp90 inhibitors and DHA or CQ is not a liability of Hsp90 inhibitors as potential components of combination therapies. Because there is an additive effect among the anti-malarial drugs and the chaperone inhibitors, and the pharmacodynamics will be complementary to those of the artemisinin derivatives [58].

A significant hurdle for using Hsp90 inhibitors as antiplasmodial agents is the perceived notion of toxicity associated with these classes of compounds. A potential liability also evidenced by the limited differential affinity between the *P. falciparum* and human Hsp90s. However, the most potent inhibitors, NVP-AUY922 and 17-DMAG, showed no toxicity against host cells at concentrations over 50 times their IC_50_ against *Plasmodium*. Despite the very comparable *K*_*d*_s against the parasite and host chaperones, 1.32 × 10^−8^ vs. 1.29 × 10^−8^ M for NVP-AUY922, and 8.23 × 10^−9^ vs. 1.58 × 10^−7^ M for 17-DMAG, respectively. But, this is not a surprising result since there is a firm body of evidence that supports the fact that Hsp90 inhibitors are well tolerated by normal cells [44]. Side effects reported for these compounds in clinical trials are mostly due to the longer treatment regimens associated with anticancer chemotherapy [50]. The limited toxicity measured for the tested compounds implies over ~130 and 70-fold difference between the parasite and host IC_50s_ for NVP-AUY922 and 17-DMAG, respectively. These differences provide an estimate of the therapeutic window available for these Hsp90 inhibitors to treat *Plasmodium* infections. Furthermore, dosage studies for 17-DMAG and NVP-AUY922 in mice have shown that doses up to 50 mg/kg are well tolerated without any visible side effects in the animals [14, 16, 29]. In the case of the resorcinol compound, this dosage reaches a C_max_ of 35 µM and an AUC of 15 µM in plasma [29]. Those values are at least two orders of magnitude higher than the necessary concentration to obtain a maximal cytocidal effect against *P. falciparum* in the recrudescence assay.

**Fig. 5:**
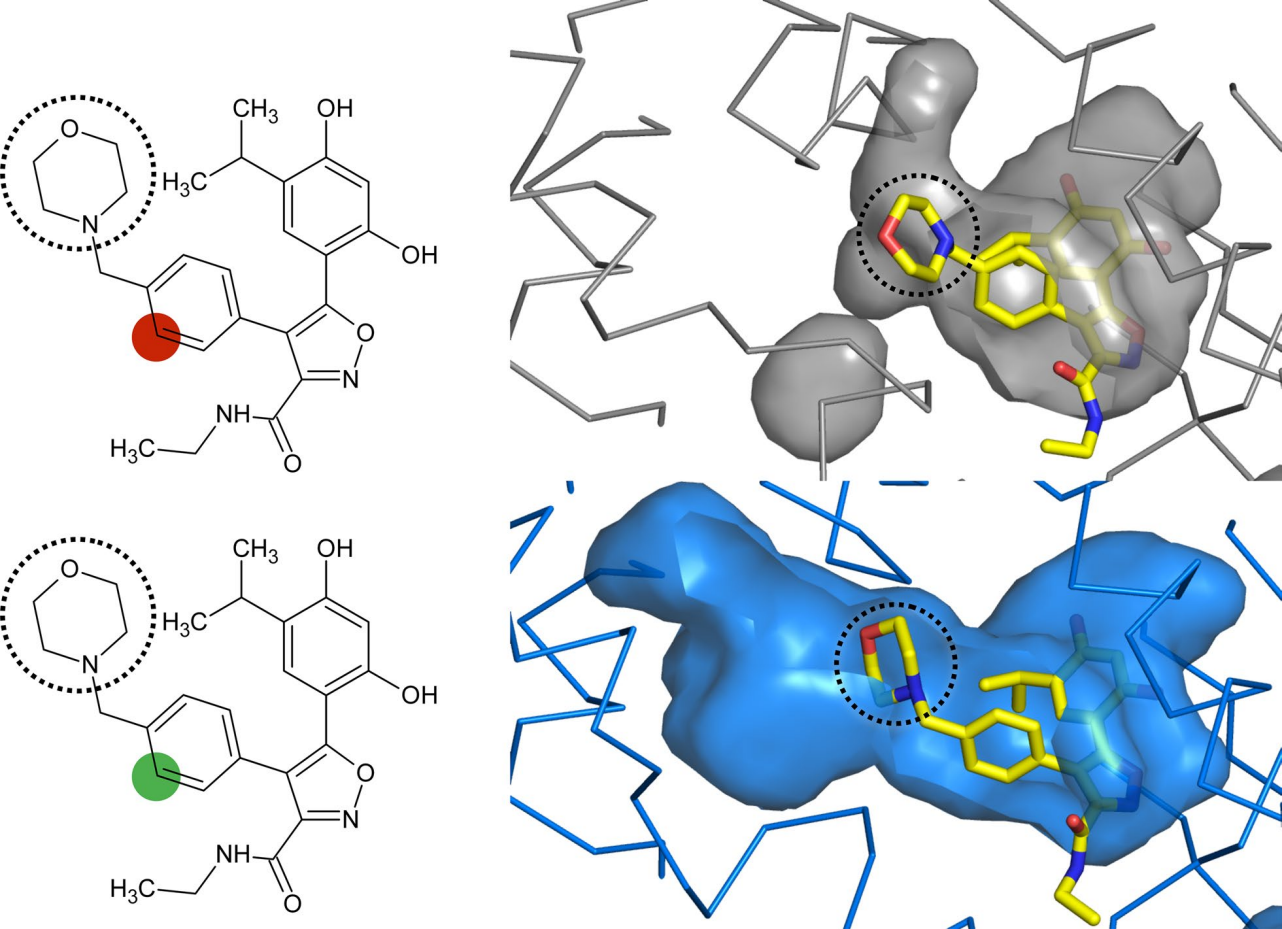
The ATP binding site of the human (*grey*) and *P. falciparum* (*blue*) Hsp90. A surface representation of the nucleotide-binding pocket for both structures is shown with the Hsp90 inhibitor NVP-AUY922 bound. The human is derived from a complex x-ray structure (PDB 2VCI [29]) and the *Plasmodium* is a docking of the Hsp90 inhibitor in the x-ray structure (PDB 3K60 [36]). The NVP-AUY922 is depicted on the *left* with a highlighted position that can be modified to take advantage of the differences in the pocket

Notwithstanding the low toxicity, generating *Plasmodium* specific inhibitors will improve the therapeutic index for Hsp90 inhibitors against malaria. The Hsp90 ATP-binding domains of *P. falciparum* and its human host share 75% sequence identity and a similar three-dimensional structure (r.m.s.d. 0.79 Å over equivalent Cα atoms) [36]. Despite this similarity, however, a close analysis of their ATP binding sites reveals significant differences between the two chaperones’ binding sites [59, 60]. The *P. falciparum* nucleotide-binding site has an extended pocket that is absent in the human chaperone (Fig. 5). This parasite-specific pocket provides a feature that NVP-AUY922 derivatives could take advantage to generate parasite specific Hsp90 inhibitors. These derivatives will be expected to be specific for *P. falciparum*, and poor binders of the human Hsp90.

## Conclusions

To summarize, it has been shown that repurposing Hsp90 inhibitors from the anticancer field represents a viable alternative for identifying antiplasmodial compounds. A diverse set of compounds was screened, and two 17-DMAG and NVP-AUY922 emerged as clear lead candidates to develop specific inhibitors against *P. falciparum*. NVP-AUY922 is favored over the geldanamycin derivative because, this scaffold has encountered limited success in clinical trials [18], consequently tempering the interest in these compounds. Additionally, the results clearly showed the resorcinol compound potent antiparasitic activity against both, the CQ-sensitive and the CQ-resistant *P. falciparum* strains. Finally, this scaffold shows a great potential to develop parasite specific inhibitors. In conclusion, NVP-AUY922 an Hsp90 inhibitor in clinical development represents a very promising scaffold for developing an anti-malarial drug.

## Additional file

**Additional file 1: Figure S1.** Detailed information regarding dose response curves. **Figures S2–S4.** The concentrations used in the dose matrix combinations and the results of these studies. **Figures S5–S7.** The Biacore sensorgrams.
