# Supplementary material for: Identification and characterization of the antiplasmodial activity of Hsp90 inhibitors
Source: Malar J. 2017 Jul 19;16:292. doi: 10.1186/s12936-017-1940-7 (PMC5518105; doi:10.1186/s12936-017-1940-7)
Supplement: Supplementary file 1 — Additional file 1: Figure S1. Detailed information regarding dose response curves. Figures S2–S4. The concentrations used in the dose matrix combinations and the results of these studies. Figures S5–S7. The Biacore sensorgrams. [file 12936_2017_1940_MOESM1_ESM.pdf]

SUPPLEMENTARY INFORMATION

Identification and characterization of the antiplasmodial activity of Hsp90 inhibitors

Claribel Murillo-Solano, Chunmin Dong, Cecilia G. Sanchez and Juan C. Pizarro

Figure S1

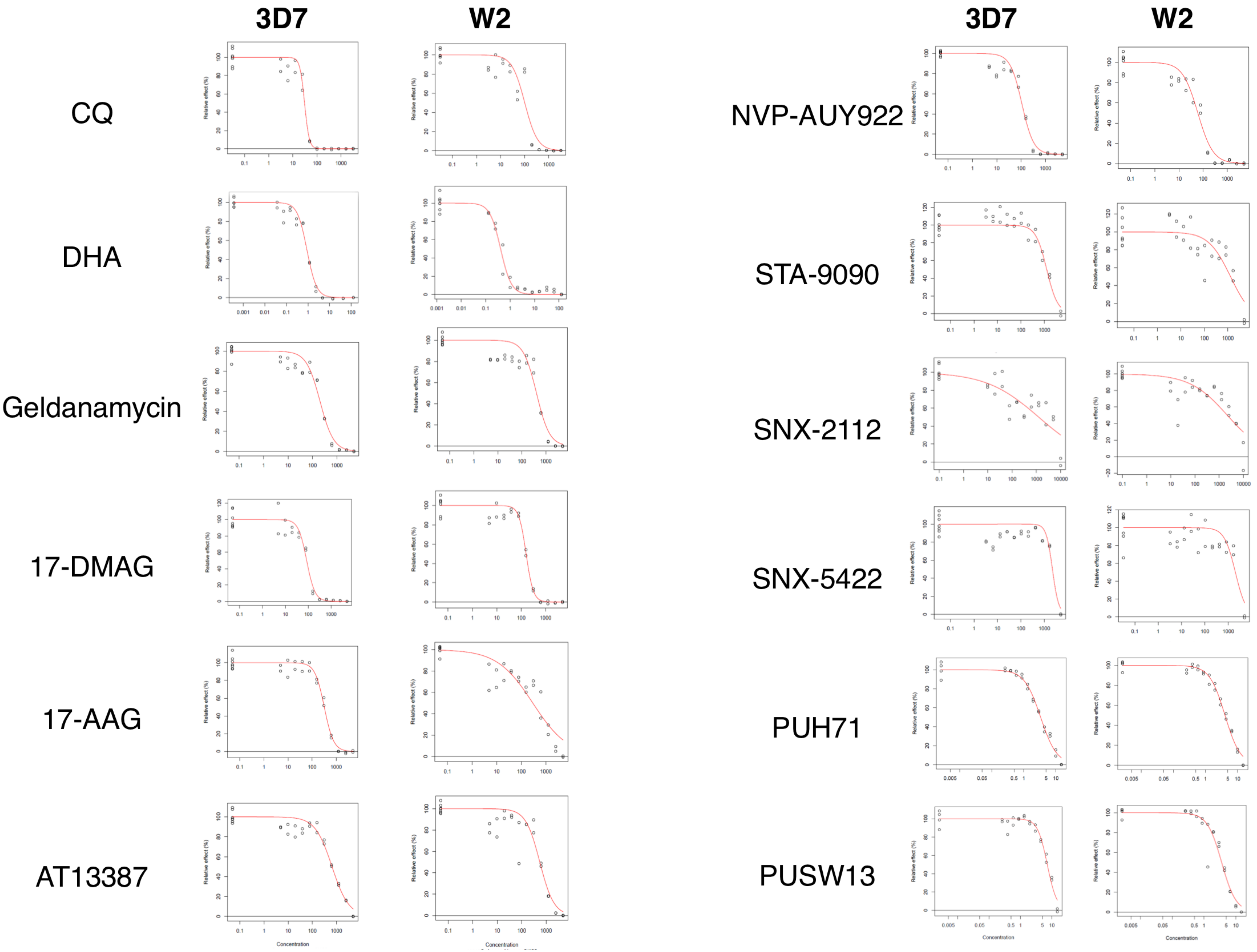

Figure S1. Dose response curves of different Hsp90 inhibitors against *P. falciparum* 3D7 and W2.

Figure S2

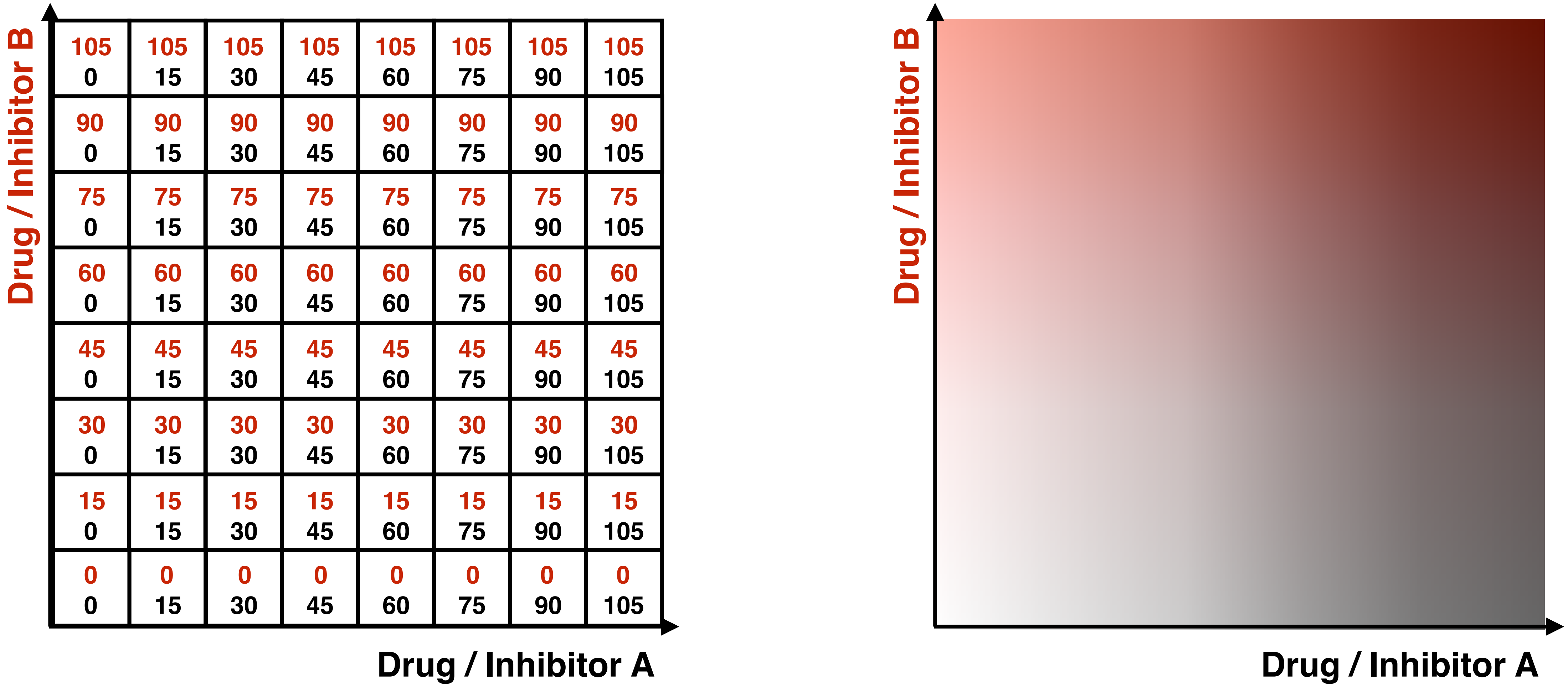

Figure S2. Plate configuration for the Hsp90 inhibitor / antimalarial drug interaction assays. The concentrations on the left are expressed as a percentage of the IC_{50} of the compound or antimalarial drug.

Figure S3

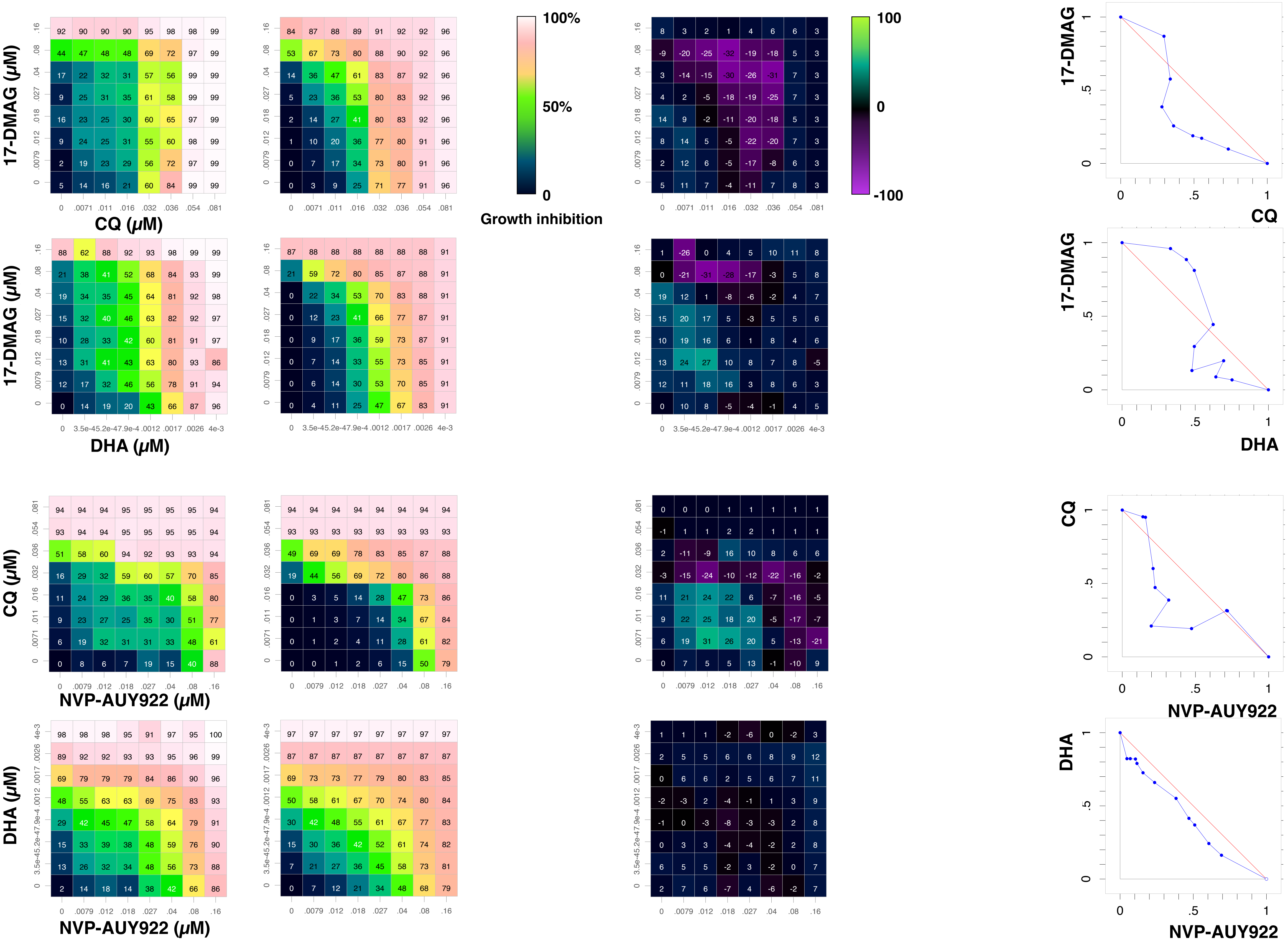

Figure S3. Hp90 and antimalarial drug combination assays for *P. falciparum* 3D7 strain. On the left the average of two growth inhibition experiments, the Loewe additivity expected values and the differences between the observed data and the model. On the right isobologram obtained with the isobole line in red. The area below the red line represent synergistic interaction, above antagonistic and around it no-interaction (additive).

Figure S4

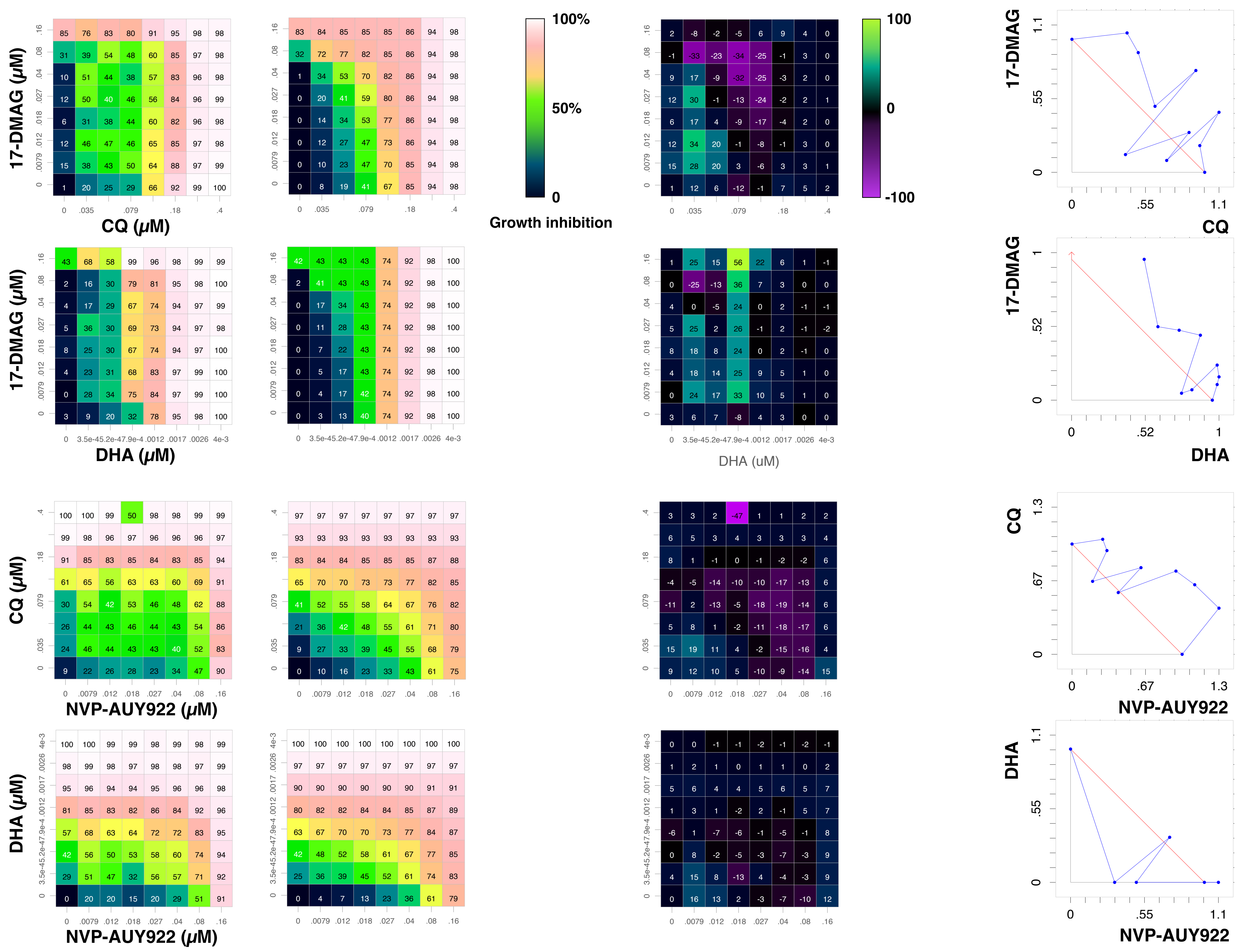

Figure S4. Hp90 and antimalarial drug combination assays for *P. falciparum* W2 strain. On the left the average of two growth inhibition experiments, the Loewe additivity expected values and the differences between the observed data and the model. On the right isobolograms obtained with the isobole line in red. The area below the red line represent synergistic interaction, above antagonistic and around it no-interaction (additive).

Figure S5

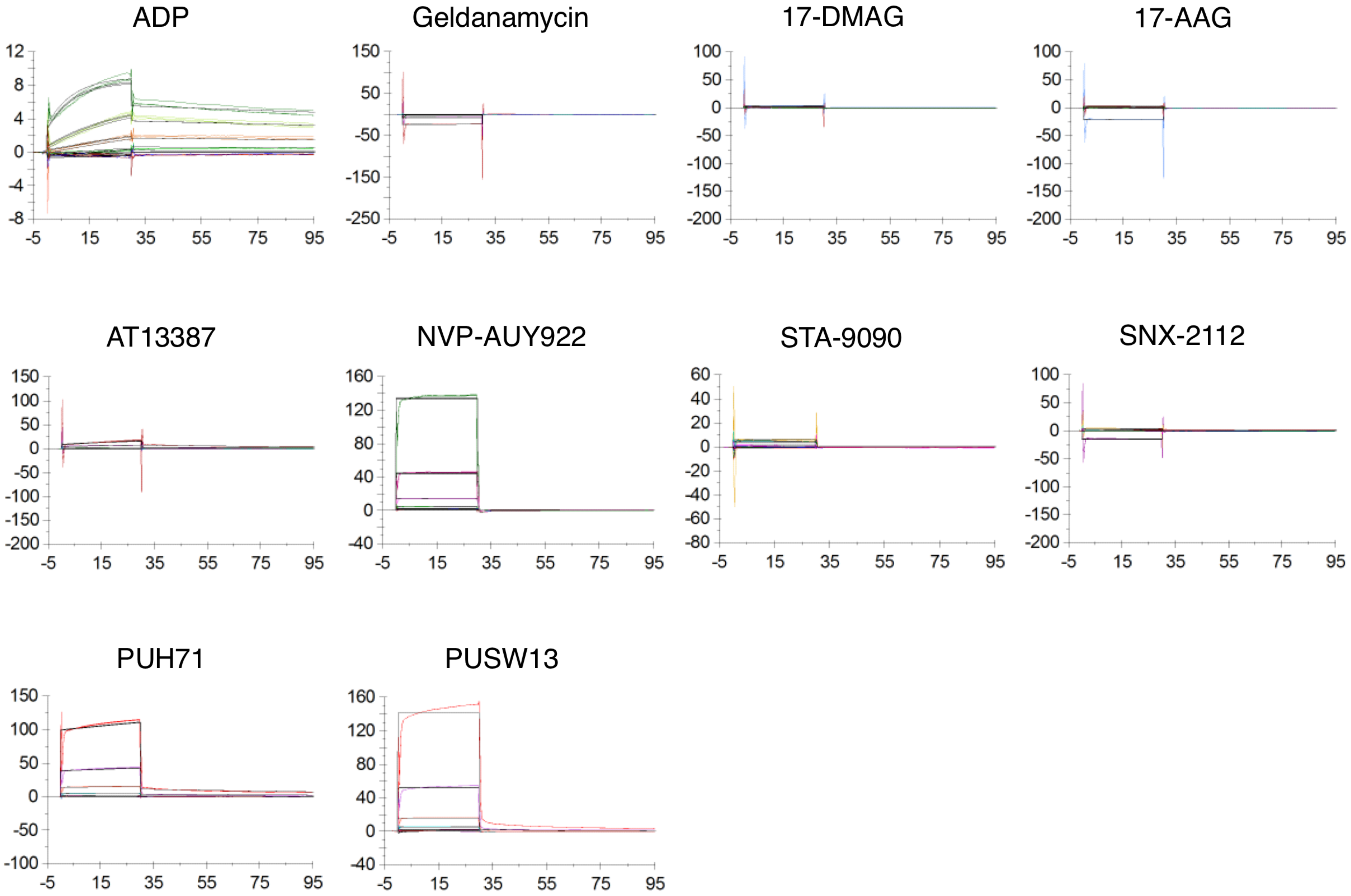

Figure S5. *P. falciparum* Hsp90 sensorgrams of the interaction between the parasite NTD against ADP and Hsp90 inhibitors.

Figure S6

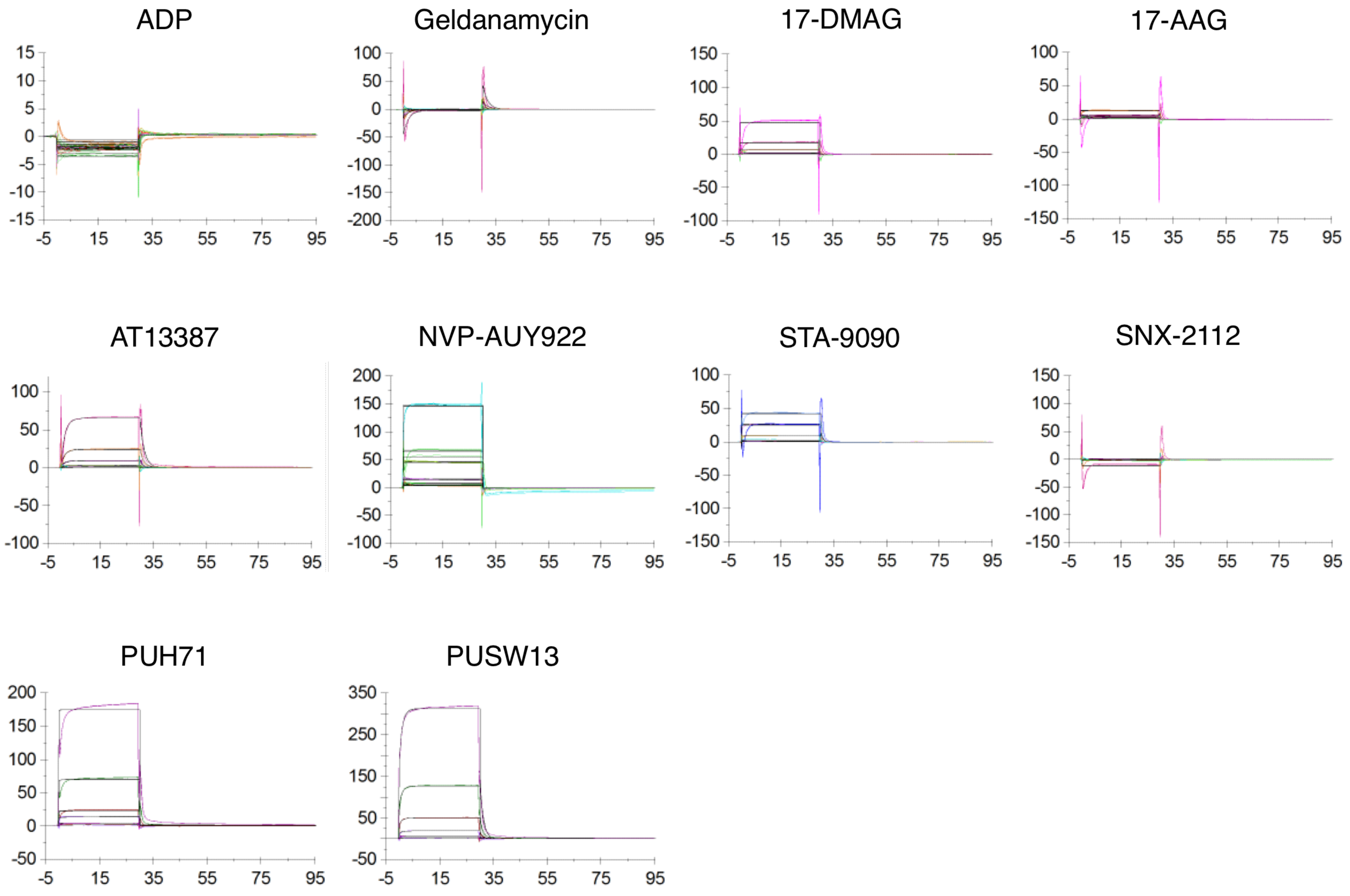

Figure S6. *P. falciparum* Grp94 sensorgrams of the interaction between the parasite NTD against ADP and Hsp90 inhibitors.

Figure S7

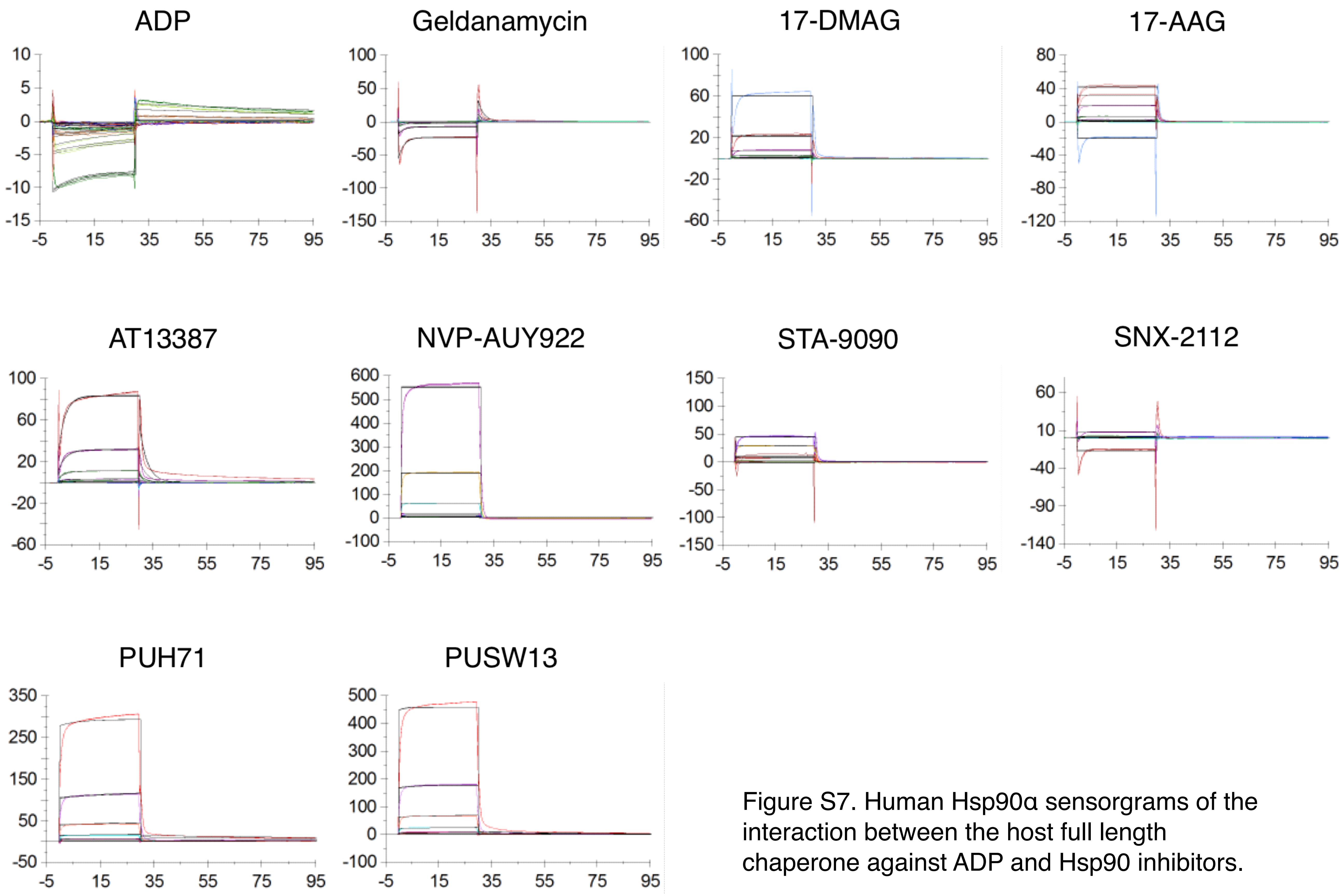

Figure S7. Human Hsp90α sensorgrams of the interaction between the host full length chaperone against ADP and Hsp90 inhibitors.
